# Evidence of diet, deification, and death within ancient Egyptian mummified animals

**DOI:** 10.1038/s41598-020-69726-0

**Published:** 2020-08-20

**Authors:** Richard Johnston, Richard Thomas, Rhys Jones, Carolyn Graves-Brown, Wendy Goodridge, Laura North

**Affiliations:** 1 Advanced Imaging of Materials (AIM) Facility, College of Engineering, Swansea University, Swansea, SA1 8EN UK; 2 School of Archaeology and Ancient History, University of Leicester, Leicester, LE1 7RH UK; 3 School of Biosciences, Cardiff University, Cardiff, UK; 4 The Egypt Centre, Museum of Egyptian Antiquities, Swansea University, Swansea, SA2 8PP UK

**Keywords:** Imaging techniques, Archaeology

## Abstract

The clues to life and death of mummified animals can remain hidden beneath their wrappings. Developments in non-invasive imaging have enabled detailed study of their internal structures. Laboratory-based X-ray microcomputed tomography (microCT) and focussed imaging protocols permit smaller mummified remains, such as animals, to be studied at higher resolution. In this study, we use microCT to image three different animal mummies. Revealing the internal structures provides insights into their biography, the conditions in which they were kept, complex mummification practices, possible causes of death, and subsequent handling damage. Thousands of years after the production of these mummified animals, the X-ray microCT technique facilitates new investigations, revealing ‘harder’ skeletal structures, mummification materials, and even desiccated soft tissues. Potential evidence for an ‘opening of the mouth’ procedure was found in a snake, along with indicators of the poor conditions in which the snake was kept when alive, leading to dehydration. Examination of a cat mummy revealed it was less than five months old and had its neck purposefully broken. It was also possible to identify a bird mummy to species level from the X-ray data. Improved understanding of animal mummification through scientific imaging can thus inform conservation and understanding of past human-animal relationships.

## Introduction

Ancient Egyptians mummified their human dead to ensure their rebirth in the afterlife. Human mummification is known throughout the Pharaonic period. In addition to humans, animals were mummified, including cats, ibis, hawks, snakes, crocodiles and dogs. Ikram^1,2^ has suggested that mummified animal remains can be divided into six categories: {1} pets buried with their owner; {2} victual mummies buried with the human to provide food in the afterlife; {3} sacred animals which were worshipped during their lifetime; {4} votive offerings which depicted the gods and were placed in temples as offerings; {5} false/amalgam; and {6} other.

Votive offerings are by far the most common animal mummies. Their production began in earnest in the Late Period (672-332 BC) and continued into the Roman Period, at least through to the fourth century AD, when they number in the millions^3^. Votive offerings were given to gods, with particular animals associated with specific deities. Gods could also be symbolised as animals, such as the goddess Bastet, who could be depicted as a cat or other feline, or a human with feline head; and the god Horus who was often depicted as a hawk or falcon^1,4,5^. Mummified animals were purchased by visitors to temples, who, it has been suggested, would offer them to the gods, in a similar way that candles may be offered in churches today. Egyptologists have also suggested that the mummified votive animals were meant to act as messengers between people on earth and the gods^1,6^.

The animal mummification ‘industry’ required high production volumes, necessitating significant infrastructure, resources, and staffing of farms that reared animals for mummification and subsequent sale^7^. Dedicated keepers were employed to breed the animals, while other animals were imported or gathered from the wild. Temple priests killed and embalmed the animals so they were made suitable as offerings to the gods^6,8^.

Since at least the 1800s, animal mummies have been studied using intrusive or non-intrusive techniques. Intrusive techniques usually involve unwrapping the mummy, revealing the bones and other artefacts that are placed inside. These techniques can provide information on wrapping techniques^9^, while chemical analyses can be used to reveal information about the mummification processes^10^. However, these techniques destroy or disturb the mummy to some extent; consequently, non-invasive procedures have been increasingly favoured. These include studying the wrappings through simple observation and polarised light microscopy, together with literature from ancient texts^6^. Pertinent to this study is the application of conventional radiography^11–16^, and medical X-ray computed tomography to mummified remains^5,12,17–30,30–37^.

Such methods are not unproblematic, however. While conventional (2D) radiographic image capture and analysis requires less commitment in terms of time and computational power, the image is a projection through the entire contents of the mummy. Consequently, it can be difficult to determine the exact three-dimensional position and orientation of the contents of mummies due to flattening and foreshortening. This is especially problematic for animal mummies because the limbs tend to be folded inwards for wrapping and mummification. This can lead to interpretative uncertainty and bone measurement errors.

Medical CT has advantages over 2D radiography, particularly in providing information concerning the three-dimensional location of artefacts and body parts^38^. However, the relatively low-resolution limits the level of insight that can be drawn, particularly in relation to the identification of features in smaller animals. X-ray microCT has been used for higher resolution studies of a mummified falcon, identifying a possible last meal^39,40^, and on smaller parts of a human mummy (a severed hand)^41^.

In this study, we describe the advanced application of X-ray microCT scanning to a range of mummified animal specimens of varying shape, size and mummification methods to provide unique insights into animal mummification practices in Ancient Egypt. MicroCT is used extensively within materials science to image internal structures on the micro-scale. The X-ray microCT method builds a 3D volume (or ‘tomogram’^42^ from many individual projections or radiographs. Hundreds or thousands of individual 2D X-ray projections are sampled at the detector while the specimen rotates between the fixed X-ray source and detector. The X-ray projections are 2D greyscale images based on the attenuation of X-rays through the sample material. A tomogram is reconstructed from the 2D images using algorithms specific to the experimental setup, consisting of a 3D matrix of isotropic voxels. Each voxel is assigned a grey value derived from a linear attenuation coefficient that relates to the density and atomic number of materials being scanned. MicroCT imaging typically has a spatial resolution a hundred times smaller than medical CT^43^, therefore enabling 3D imaging and analysis of smaller internal features. The spatial resolution in microCT imaging is also related to the width of the specimen. Wider specimens typically result in lower spatial resolution because successful filtered back-projection reconstruction of the 3D data requires the entire sample width to be encompassed within each 2D projection or ‘field of view’ at all rotations^44^, therefore requiring a lower magnification or larger source-sample distance. A typical X-ray detector panel in a laboratory microCT setup has a width of around 1,000–4,000 pixels. For a detector of width 2000 pixels, the pixel size (and ultimately 3D voxel size of the reconstructed tomogram) is at best w/2000, where w is the maximum width of the specimen. The spatial resolution is typically a few pixels^42^, and is therefore limited in this method by the width of the specimen. Rueckel et al. have also shown that in addition to magnification and specimen size, spatial resolution is a function of X-ray tube current and voltage^45^.

We use X-ray microCT and a region of interest scanning modality to image the internal structures within Egyptian mummified animals in three dimensions and at micro-resolution. We highlight the additional level of detail that can be observed and the insight this can provide in resolving the biographies of the animals, their cause of death, mummification practices, and subsequent handling damage. We pioneer a Region of Interest (ROI) scanning technique to provide higher resolution imaging of the internal parts of such specimens. The traditional methodology in microCT scanning requires the entire specimen to remain in the field of view for all projections. We demonstrate that for animal mummies, where identification and measurement of critical internal features provide important information, this can be circumvented by zooming into internal parts of interest within the specimen to acquire projections at a higher resolution.

## Materials

**Figure 1:**
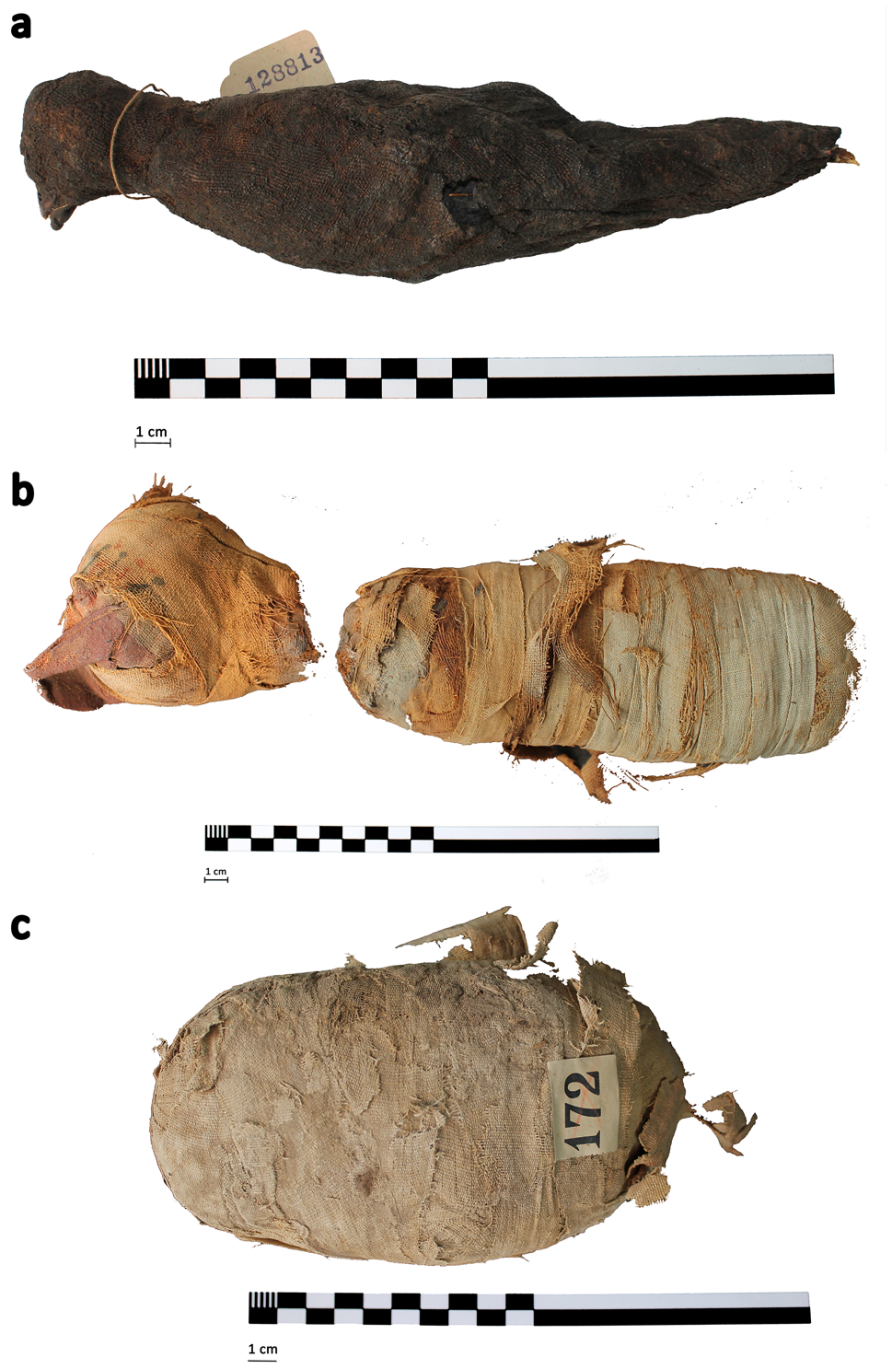
Photographs of all three specimens and scale bars: **a** bird mummy (W531), **b** cat mummy (head: AB77a and body: AB77b), and **c** mummified snake (EC308).

Specimens were selected from the collection at the Egypt Centre, Swansea University. To demonstrate the capability of X-ray microCT for high-resolution, non-destructive investigation of three mummified animal remains of varying size and shape: a bird, a cat, and a snake (Fig. 1). The specimens were relatively well preserved externally, but the condition of the internal structures was unknown prior to imaging.

The mummified bird of prey (W531) (Fig. 1a), was purchased by Sir Henry Wellcome from the auction rooms of JC Stevens, Covent Garden, London, on 15th October 1930 for 21 shillings (lot 578). The Egypt Centre has no record of where the auction items originated; very often auction houses did not record provenance at this date. The item was given on long-term loan to Swansea University (then University College of Swansea) in 1971 by the Wellcome Trustees. It is relatively intact externally, except for a leg protruding from the bottom, which has been severed at the midpoint of the tarsometatarsus and is missing the foot.

The cat mummy is composed of two specimens with the head (AB77a) separate from the body (AB77b). The consistency of wrapping between the head and body suggests this separation occurred after mummification. The head is decorated with a painted burial mask (Fig. 1b). AB77a and AB77b were donated to the Egypt Centre at Swansea University in 1997 by the School of Art at the University of Aberystwyth. Joseph Davies Bryan had donated the specimens to Aberystwyth. As Bryan lived and worked in Cairo, it is possible the cat mummy originated from that area. Cat mummy cemeteries have also been found at Bubastis, Thebes, Saqqara, and Beni Hasan.

Whilst it is sometimes possible to determine the animal from the shape of the mummified specimen, they can be difficult to identify. One example of such a specimen is labeled EC308 and was accessioned as ‘mummified animal, possibly human’. The object is an oval package, tightly wrapped in linen bandages (Fig. 1c). It bears an old serrated paper label with the number ‘172′ printed thereon, and a catalogue card in the Egypt Centre suggests that the item with this number is from the Rustafjaell collection. The paper label looks like a cataloguing number and was likely purchased by Sir Henry Wellcome from the 1906 Robert de Rustafjaell collection sale. In 1971 they were loaned to Swansea University by the Wellcome Trustees. In 2009, a 2D radiograph carried out at a local veterinary clinic revealed the contents to be a coiled mummified snake, but further analysis was not possible using only 2D X-ray imaging.

## Results

### Cat head (AB77a)

**Figure 2:**
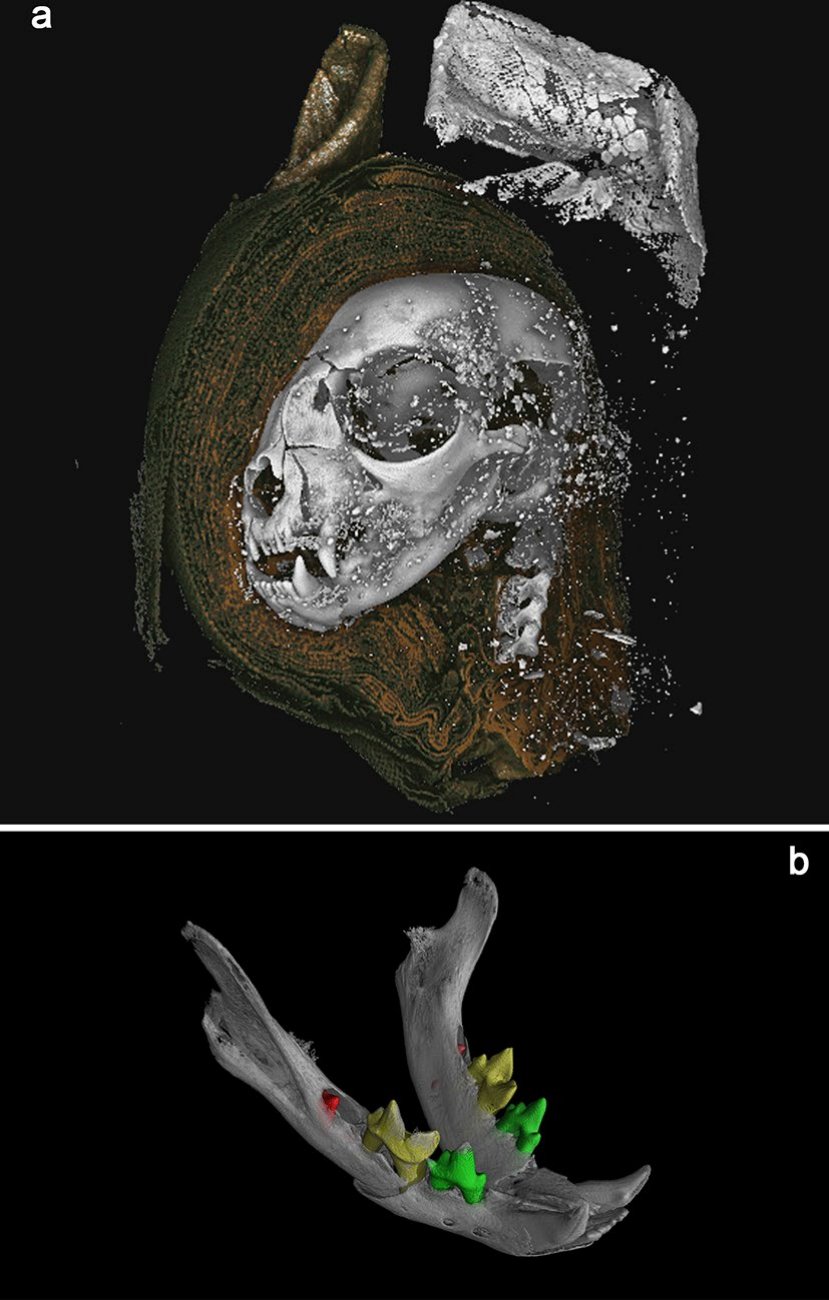
3D renderings from microCT data. **a** Mummified cat head (AB77a) rendered from tomography data. A digital dissection, removing wrappings on left side of the head, revealing bone, and higher attenuating material used to stiffen the external wrapping of the ears. **b** Cat Head (AB77a) mandible, with segmented teeth, revealing unerupted mandibular first molars (red). Scale: skull total length = 68.9 mm.

The scans of the cat head produced a reconstructed volume with a voxel size of 78.1 µm. On the macro scale, the skull of the cat is clearly much smaller, around half the size of the external mummified wrappings (Fig. 2a), which can be digitally unwrapped through segmentation of the greyscale data to reveal the bone (Supplementary Information Movies S1 (10.6084/m9.figshare.12328301) and Movie S2 (10.6084/m9.figshare.12328301)).

Four members of the *Felis* genus exist in Egypt: domestic cat (*Felis catus* Linnaeus 1758), wildcat (*Felis sylvestris* Schreber 1777), swamp cat (*Felis chaus* Güldenstaedt 1776) and sand cat (*Felis margarita* Loche 1858). Amongst Egyptian felids, *F. chaus* is considerably larger than *F. sylvaticus libyca*, which is in turn larger than *F. margarita*^46^. While there is some overlap, *F. sylvaticus* is generally larger than its domestic counterpart (*F. catus*)^47^. Despite the high-resolution precise data generated via microCT, accurate measurements of AB77a are hampered by the damage to the cranium and the young age of the individual (described below). Nevertheless, comparison with Egyptian felids and domestic cats from the former Czechoslovakia reveals that an attribution of *F. catus* is most likely (Supplementary Information Table S1).

Analysis of dentition indicates that the cat was less than five months old at the time of death. The deciduous premolars are present within the mandible, which erupt around 5–6 weeks and are replaced around 4–5 months^48^. The first molars, which erupt around 130 days^49^, are unerupted and located within the alveolar crypt (Fig. 2b and SI Movie S3 10.6084/m9.figshare.12360674).

**Figure 3:**
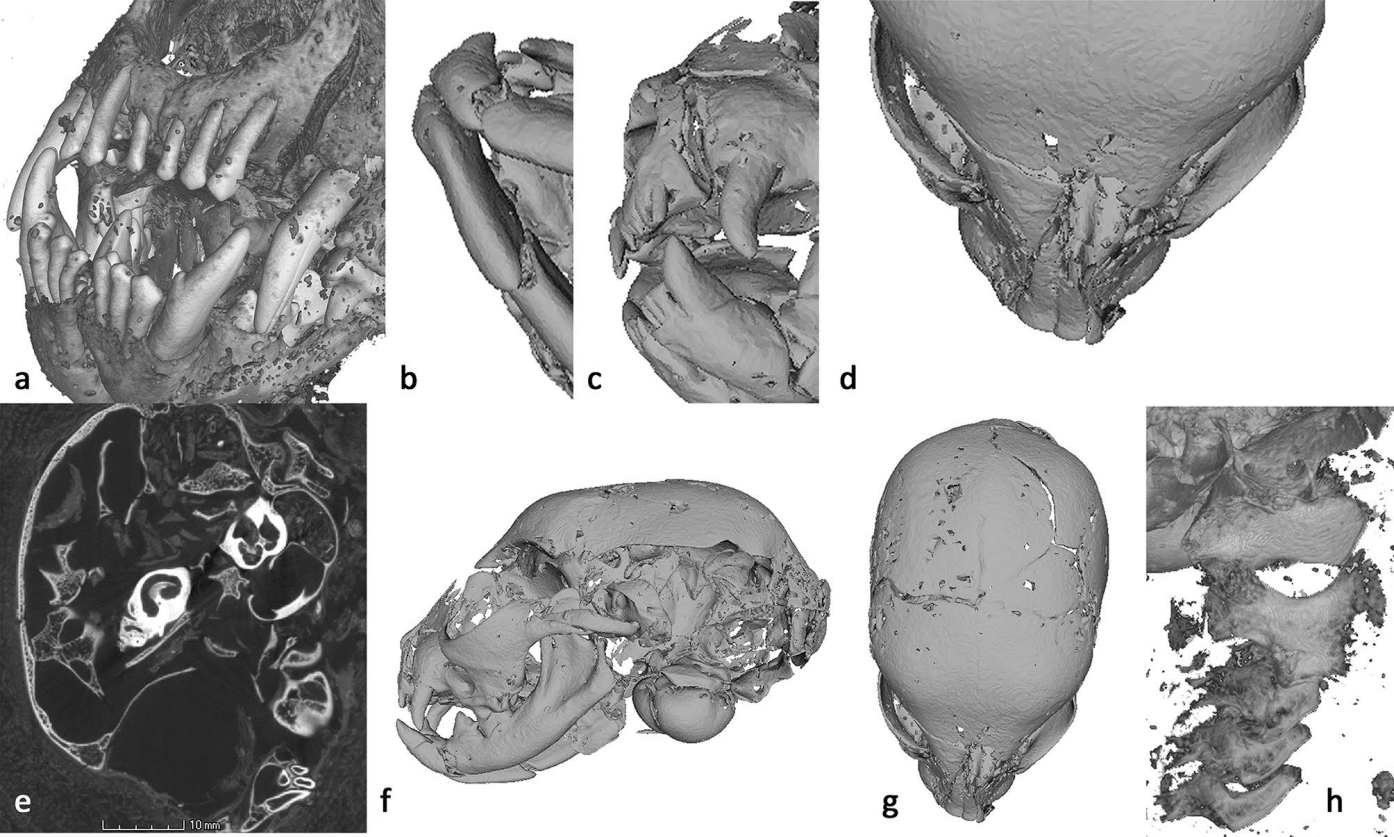
Cat Head (AB77a) microCT visualization—**a** dentition, **b** mandibular fractures, **c** left maxilla fracture, **d** angular deviation of the nasal, **e** fragmentary parts of the cranial wall and internal structures of the ear visible within the cranial cavity (2D slice image from the tomogram), **f** radiating fracture across left parietal, **g** radial fracture terminates at the suture lines, **h** atlas, axis, and cervical vertebrae, indicating separation and possible cause of death. Scale: skull total length—68.9 mm.

Oblique fractures are evident in both mandibles (Fig. 3b), with minimal displacement. On the right side, the fracture is located between the third and fourth deciduous premolar (Fig. 3a); on the left side the fracture is located marginally anterior to the third deciduous premolar (Fig. 3c). The fracture is oriented in the same direction in both mandibles—diagonally posterior to anterior—indicating that it occurred in the same event; the angle and direction of displacement is suggestive of a powerful impact from below the mandibles. The absence of bone healing suggests that the impact occurred at or after the time of death.

Trauma is apparent in the left maxilla anterior of the canine (Fig. 3c) and probably caused the angular deviation of the nasal (Fig. 3d) and radiating fractures of the left maxilla. The right maxilla appears unaffected. Large portions of the left and under side of the skull are absent including the distal portion of parietal, squamous part of temporal, basi-sphenoid, basi-occipital, part of the tympanic bulla and internal structures of the ear (Fig. 3e). The fact that two fragmentary parts of the cranial wall and parts of the petrous are present within the endocranium would suggest that this trauma occurred post-mortem, after the brain had decomposed.

A radiating fracture runs across the left parietal, caused by blunt force trauma to the left side of the cranium and resulting in the loss of these parts of the skull (Fig. 3f). Fleming-Farrell et al.^50^ have identified five criteria by which peri-mortem and post-mortem blunt force trauma to the cranium can be separated reliably: preponderant texture; preponderant outline; relationship to the path of least resistance; plastic response; and the presence of hinging. The absence of a plastic response and hinging, the rough texture, and irregular outline (Fig. 3f,g) all point to post-mortem damage. Furthermore, ante-mortem fractures in living individuals often track along the suture lines (because they are structurally weaker), with radial fractures crossing suture lines to the adjacent plate(s)^51^. In contrast, radial cracks tend to terminate at the suture lines in post-mortem fractures of dry bone^51^, which is the case in AB77a (Fig. 3g). Taken together, this evidence supports the view that the damage to the left side of the skull occurred post-mortem.

The atlas, axis, and three cervical vertebrae are included with AB77a. As AB77a (head) and AB77b (body) are separate, it is not possible to determine if the vertebral separation between samples was the cause of death, or if it occurred during mummification or later. Further analysis of AB77a reveals a separation of 5.98 mm between the axis and the atlas vertebrae (Fig. 3h) providing clues to a possible cause of death.

Residual brain matter is evident within the cranium (Fig. 3e). The fact that this is on the right-hand side towards the back possibly indicates the position of the mummy following mummification, or the scanning position if they are loose.

Efforts could be made to ‘retrodeform’ or virtually reconstruct the cranium to near its original configuration from the detailed microCT data, similar to processes used to identify damaged fossil hominid specimens^52–54^. It is possible this would then allow more accurate morphometric analyses of the skull, and a determination of the species, but this would still be challenged by the young age of the cat.

Within the visualization software, it was possible to segment the skull from the desiccated soft tissues and wrappings based on its X-ray attenuation/greyscale value, and to export a surface in stereolithography file format (.STL). This is a common format for 3D printing, which typically has a much smaller file size than the 3D tomogram data (52 MB compared to 2.4 GB). STL file types can be viewed, rotated, manipulated, and sectioned in many 3D visualization software packages, including freeware and open software. The smaller file size allows researchers without access to powerful computation, and who are not based within a microCT laboratory to perform visualization and analysis. An STL file is a simplified representation of the tomographic data, only representing a surface applied to a specific voxel attenuation value for bone. Therefore, representation of tomographic data via STL files facilitates morphological analyses, but does not permit analyses of contrast and varying density/attenuation.

**Figure 4:**
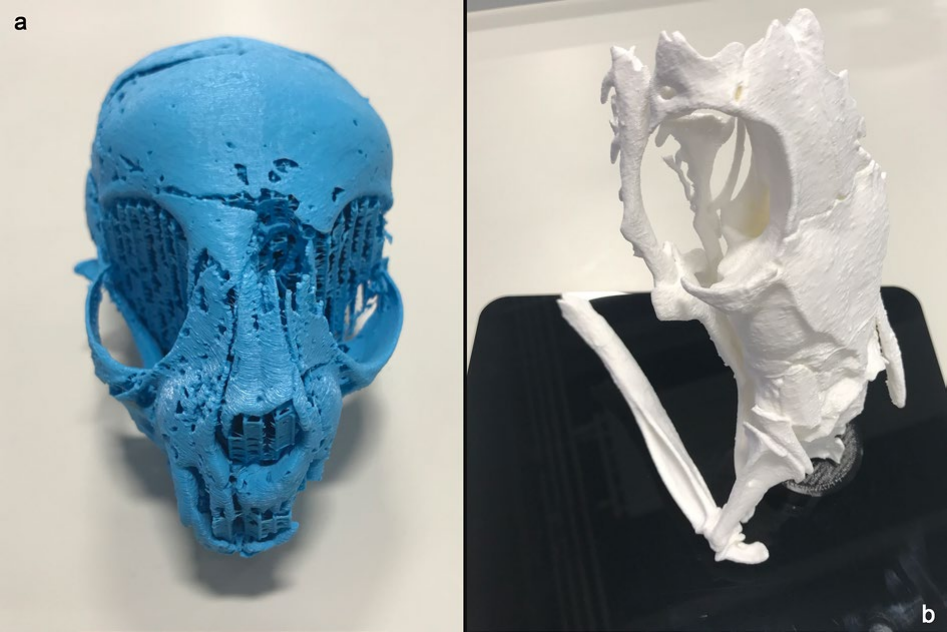
3D-prints from segmented X-ray micro tomography data: **a** skull of AB77a cat mummy, printed ~ 2.5 × larger, **b** skull of EC308 snake mummy, printed 10 × larger. Scale: printed cat skull length = 170 mm; printed snake skull length = 140 mm.

We were able to 3D print both the cat (AB77a) and snake (EC308) skulls (Fig. 4a,b), using a standard filament material acrylonitrile butadiene styrene (ABS) on an Ultimaker2 3D printer (Ultimaking Ltd., 4191PL Geldermalsen, Netherlands). The cat model (available at 10.6084/m9.figshare.9970301) was scaled 2.5 times larger than the actual skull, and the snake (available at 10.6084/m9.figshare.9902372) scaled 10×, providing a tangible 3-dimensional model to aid identification and forensic analysis.

### Cat body (AB77b)

The scans produced a reconstructed volume with a voxel size of 65.23 µm and a large 3D tomogram size of 78.86 mm × 226.07 mm × 96.41 mm. Data size = 22.2 GB. The tail of the cat had been tucked through its folded hind legs and the fore limbs placed flat alongside the body (Supplementary Information Movie S4 10.6084/m9.figshare.12360677). There does not appear to be any evidence of insertion of materials or objects inside the body of the cat, although residual fecal appears evident (visible in X-ray slice Supplementary Information Movie S5 10.6084/m9.figshare.12360680). The young age of the cat is confirmed by the presence of unfused epiphyses. The fact that the distal epiphysis of the humerus is unfused—one of the earliest parts of the skeleton to fuse—indicates the cat is younger than 18 weeks of age^55^.

### Bird of prey (W531)

The bird of prey mummy (W531) is wrapped and covered in a black, possibly resinous, material. It is believed to be votive, consistent with other studies of similar bird mummies^5^. The specimen is 23 cm long and 7 cm wide at its broadest point near the midpoint.

**Figure 5:**
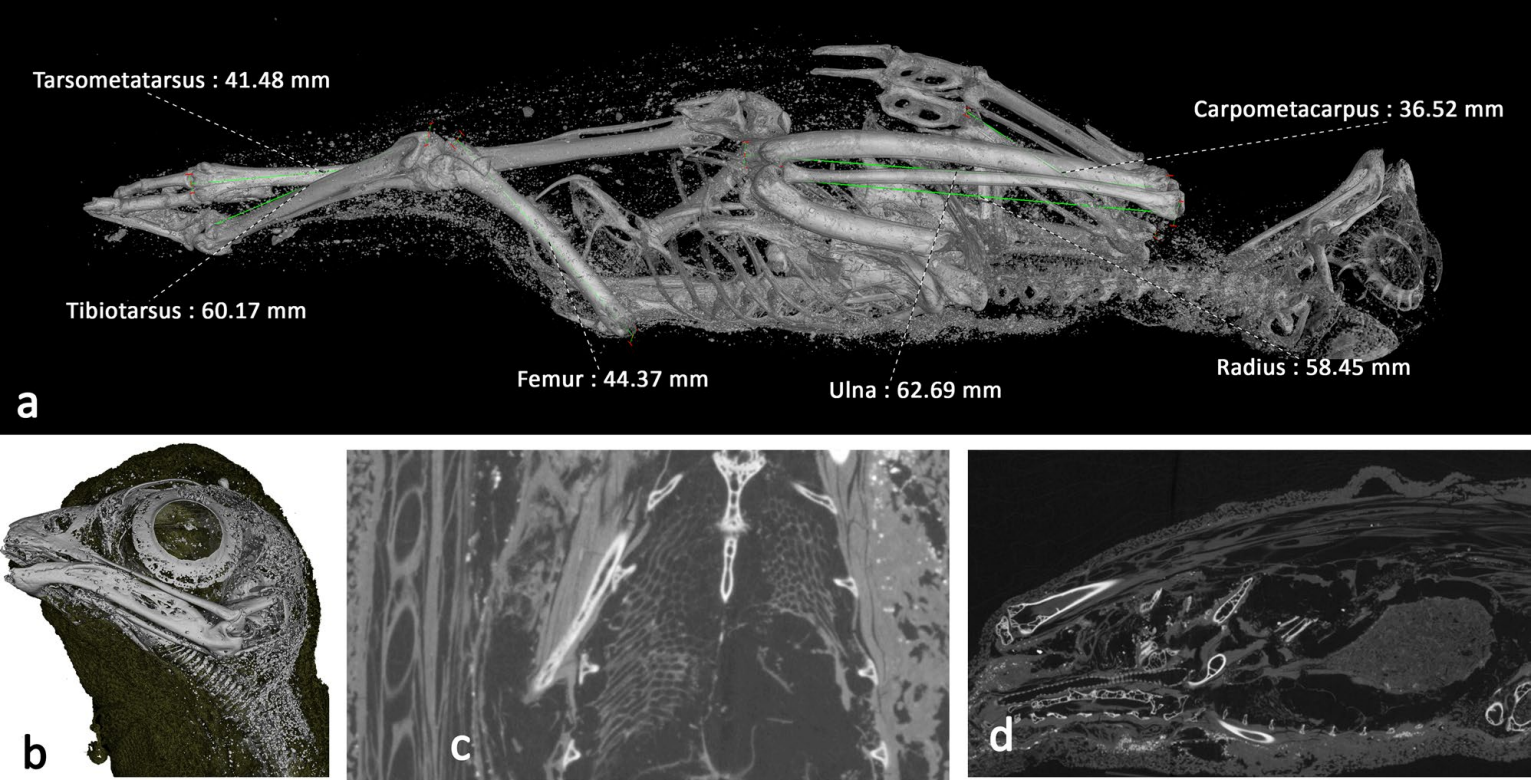
Bird of prey (W531) microCT visualisations. **a** Digital segmentation of mummified bird tomogram, revealing skeletal structure and some small higher-attenuating structures within the wrappings. Morphometric measurements of tarsometatarsus, tibiotarsus, femur, ulna, radius, and carpometacarpus are superimposed. **b** Skull with wrapping digitally removed, revealing residual trachea. **c** Coronal slice revealing internal soft tissues remain intact, including the lungs; bubble-like structures in chest cavity. The feathers are visible in the cross section as elongated ellipses to the left. **d** Sagittal slice revealing digestive system and gizzard. Spine is at the bottom of the image, top of the bird is to the left, and bottom of the bird is to the right.

External visual analysis of the remains revealed damage to the tip of the beak and damage to the left leg of the bird, although this was protruding from the wrappings, and therefore would have been susceptible to post-mummification damage. The rest of W531 appears to be superficially intact. X-ray imaging reveals the bird is 221.13 mm long, in its mummified position. Figure 5a shows a visualisation produced from the X-ray microCT data with a segmentation threshold applied to reveal the skeletal structures. To aid species identification, a number of key morphological measurements following the protocol for measuring bird and mammal bones^56^ were made of the specimen remotely and digitally, using the 3D microCT data. These are presented in Supplementary Information Table S2 along with the measurements from a selection of comparative small raptors^12,57–62^. This comparison suggests W531 belongs to the *Falco* genus, most closely resembling the Eurasian kestrel (*F. tinnunculus*).

From the microCT data and visualisations, both humerii and the left tarsometatarsus are fractured (Fig. 5a and Supplementary Information Movie S6 10.6084/m9.figshare.12360683 and S7 10.6084/m9.figshare.12411023). The oblique fracture in the left humerus is suggestive of a peri-mortem traumatic event, while the bone was still flexible. The fractured surface in the tarsometatarsus is irregular and more suggestive of post-mortem traumatic damage. There is no evidence of deviation of the cervical vertebrae associated with strangulation/neck breaking.

Tomographic slices distinguished lower attenuation structures inside the bird; structures that are not bone. One of these structures emanated from the mouth, progressed along the spine and led to the abdominal cavity of the bird. This structure, resembling a hooped tube, is the residual trachea where the cartilage has become calcified (Fig. 5b,d). The length of this is approximately 55 mm. The trachea leads into a ‘bubble’ like structure, which is visible at a similar position to where the lungs would be in a bird (Fig. 5c). The dimensions of which are 18.19 mm long, 16.76 mm wide at its broadest and 11.71 mm deep. Circular features can be seen along the outside of the animal, these are the remains of the feathers. There is another mass inside the cavity (Fig. 5d), which is 18.8 mm wide at its broadest, 32 mm high and 23.6 mm deep. This could be part of the digestive system of the animal and is likely to be the gizzard.

### Snake (EC308)

The snake specimen takes the form of an oval package, tightly wrapped in linen bandages, measuring approximately 165 × 84 × 56 mm, shown in Fig. 1c.

**Figure 6:**
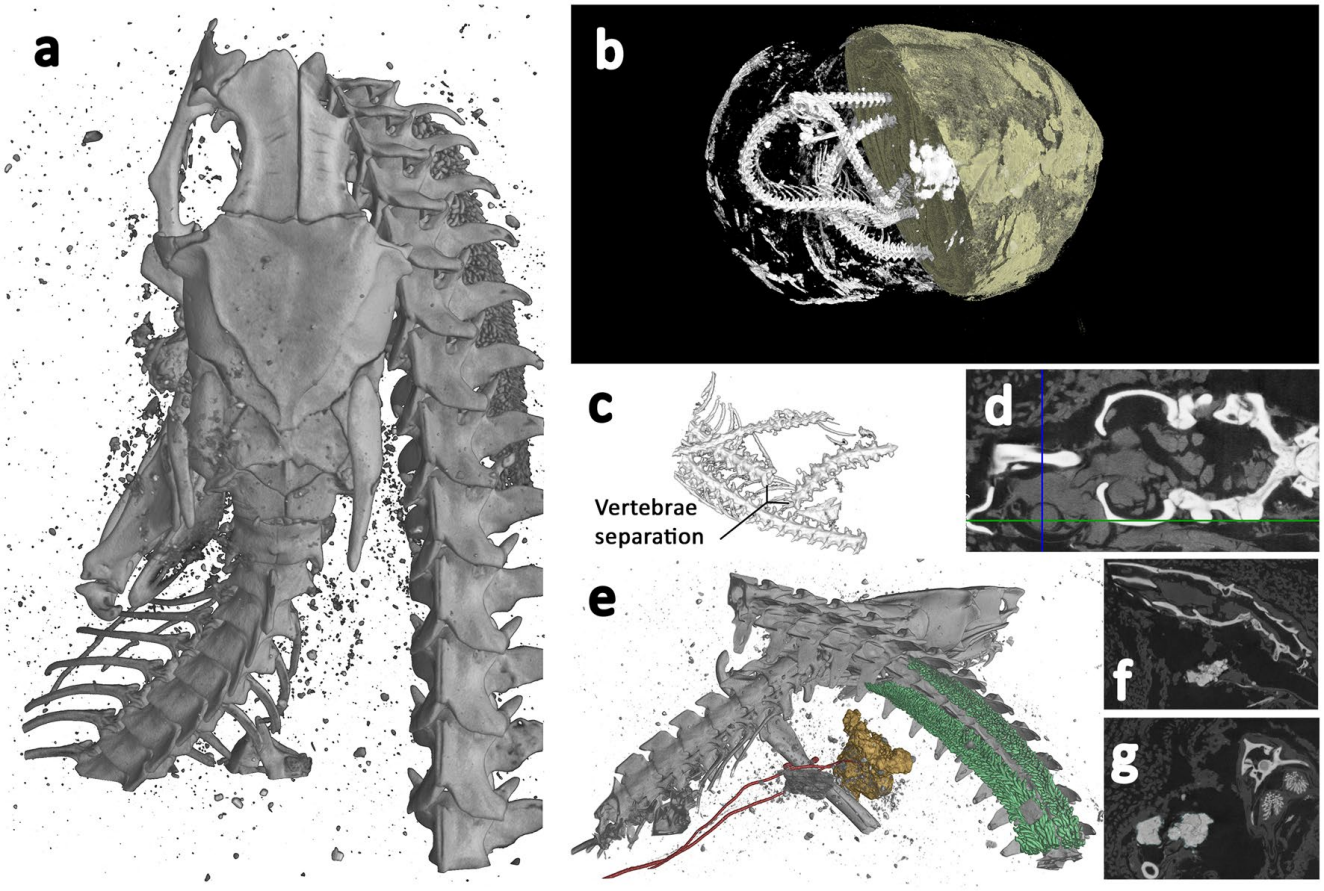
Snake (EC308) microCT visualisations. **a** Top view of 3D segmentation of ROI tomogram producing higher resolution, revealing bones with a focus on the skull and associated skeletal damage, and some calcified tissue. **b** Segmented render of whole specimen from lower resolution scan. Half of the wrappings digitally removed to reveal snake skeleton and some higher-attenuating sections within the wrappings. **c** 3D segmented sub-section of lower resolution scan showing section of separated vertebrae, with a separation of approximately 5 mm. **d** Axial slice through the skull, revealing bone (white), and desiccated soft tissue (grey) which includes residual brain matter inside the cranium and the remains of the left eye. The intersecting lines highlight the centre of the eye. **e** 3D segmentation of ROI tomogram, revealing bones (grey), trachea (red), and calcified kidneys (coloured green). **f** Sagittal slice through the skull, revealing bone (white) and structures in the mouth, possibly inserted at the opening of the glottis. **g** Coronal slice through the skull and intersecting coiled vertebrae (white), calcified kidneys (light grey), and objects possibly placed in the mouth. Scale: Overall wrapped package longest length = 165 mm, Snake skull length = 14.4 mm.

The lower resolution scan (Fig. 6b and SI Movie S8 10.6084/m9.figshare.12360689) revealed the coiled remains of a Proteroglyphous snake evident from the position and structure of the prefrontal, frontal, parietal and quadrate. Unfortunately, due to limited resolution of the whole specimen scan and several missing bones, we were unable to identify the specimen to species. However, the increased resolution of the ROI scan (Fig. 6a and SI Movie S9 10.6084/m9.figshare.12360695) revealed long cervical ribs and finer details of the skull identifying the snake as an Egyptian cobra (*Naja haje* L., 1758).

Cobras can typically be identified from their shortened maxillae that bear few teeth except for a pair of significantly enlarged downward pointing needle-like fangs. Identification of the scanned specimen was challenged by the damage to the skull (Fig. 6a), with the nasal, premaxilla, maxilla and fangs completely missing, and the remaining compound rotated with the dentary missing. The increased resolution of the 3D ROI scan gave more detail of the precaudal vertebrae and revealed the attached long cervical ribs of the cobra’s hooding mechanism.

Egyptian cobras are around 330 mm in length as hatchlings with adult animals attaining lengths of up to 2400 mm. The snake length measured from the 3D microCT data, is approximately 850 mm, indicating that the specimen was juvenile.

The whole specimen lower resolution scan (Fig. 6b) reveals vertebrae separation near the centre of its body length (Fig. 6c), displaying both separation of approximately 5 mm and misalignment, perhaps indicative of the cause of death. A large number of fractures were identified from the higher resolution ROI data, including the left supratemporal, left dentary/compound, right prefrontal and left maxilla. The nasal, premaxilla and fangs are completely missing from the package, as are the right dentary and compound, right maxilla, right palatine, right ectopterygoid, and right pterygoid. It is possible that other smaller bones are missing.

In addition to the skeleton, the higher resolution scan reveals desiccated soft tissue. Lower attenuation material is visible around the entire skeleton, and it is possible to see the left eye including the lens (Fig. 6d), but the right eye is no longer in place and may be missing from the package entirely. The hyoid bone is also visible near the mouth (Fig. 6e). Approximately 154 mm from the tip of the tail, two structures with relatively high attenuation are visible (Fig. 6e). These have a nodular appearance, and measure approximately 22 mm in length. They are aligned symmetrically within the body cavity, which is not common for paired snake organs as they are typically staggered within the body, but given their shape and position it is likely they are calcified kidneys.

Higher attenuating structures are also revealed within the mouth (Fig. 6e). These are not bone, but could have been placed there during mummification/wrapping. A previous study using low resolution conventional thin-section tomography, identified similar structures placed within the mouth of a mummified snake, and were regarded as an artifact of the mummification process. However, the imaging method used at the time did not have sufficient resolution to determine what these may be and their precise location^63^. Given the overall size of the snake, the structures within the mouth are small, measuring only 3–4 mm. The tomographic slices indicate they have a structure like dirt, clay, or possibly natron (Fig. 6f,g). Interrogation of the high-resolution data indicates that these structures are located at the opening of the trachea, the glottis.

**Figure 7:**
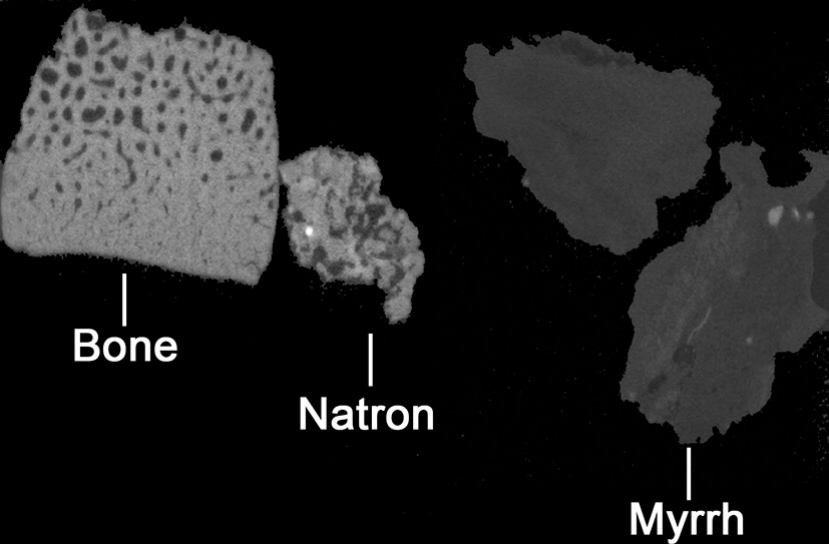
2D slices through X-ray tomograms of bone, natron, and myrrh phantom samples. Shows similar attenuation (evident in greyscale) of bone and natron, as seen in the mummified snake, EC308.

We constructed a phantom specimen from bone, natron sourced from Egypt, and myrrh to provide images for comparison to the X-ray images of the structures within the mouth of the snake. 3D tomography of this manufactured specimen shows that the items placed at the opening of the glottis are similar to small pieces of natron. This is evidenced by the similar comparative attenuation, or greyscale, of the phantom natron (Fig. 7) and the structures within the mouth (Fig. 6f,g). The digital slices through both materials also show similar shape and appearance.

## Discussion

### The mummies

Mummy AB77a and AB77b is likely to be a domestic cat, although precise species attribution is complicated by trauma and the young age of the animal. Nevertheless, this determination is consistent with previous destructive analyses of mummified animals^8,12^. Identifying species of cat within mummified packages is particularly difficult and requires accurate and precise measurement of specific bones. This is difficult to achieve with medical CT data, but is possible with the improved resolution afforded by microCT.

Large-scale trauma to different sections of the skull was identified: fractures of the maxilla and left side of the skull are confirmed to have been a consequence of storage or conditions in the thousands of years post-mummification; the oblique fractures of the mandibles could have occurred at or near the time of death, and could have contributed to the cat’s death, or perhaps be a consequence of the mummification procedure. Analysis of the teeth and epiphyses revealed that the cat was aged less than five months old at the time of death. This supports previous research^8,12^, which has shown that cats used as votive offerings were usually, though not exclusively, killed before adulthood. The separation of the axis and atlas vertebrae (Fig. 3h) indicate a possible cause of death—strangulation/neck breaking, or at the time of mummification to position the head in an upright posture. Assumed death in this way has been reported previously via 2D radiography^11,12^. However, this is the first potential example of this practice in an ancient Egyptian cat identified through 3D non-destructive means.

Using RoI imaging, package EC308 was identified as a cobra; an important snake in ancient Egypt. The cobra can represent the fiery goddess, channeling the power of the sun, illuminating the night, and effectively destroying enemies^64^ and acting as creational beings. Cobras were also associated with solar deities, such as those goddesses who were the daughters of the sun god, and with primeval (creational) gods such as Atum. Two uraei from the tomb of Rameses VI spit into receiving hands, which is a gesture of creation^65^. The uraeus is the rearing cobra often depicted on the brow of the king, and can be personified as a daughter of the sun-god. The spitting cobra, while feared, could thus be protective and creational.

The cobra identified in EC308 could have been killed by spinal fracture, evidenced by the dislocated vertebrae. This would have been sufficient to cause death in the animal and is consistent with a tail capture and ‘whipping’ method commonly used to kill snakes. This practice was also identified in a mummified cobra in the Egyptian Museum (Cairo) collection^16^. The ‘whipping’ may also have fractured the skull on impact to ensure the animal’s death. This is supported by extensive damage to the right side of the skull and missing nasal, premaxilla, maxilla and fangs. Damage to the skull and several fractured bones that are missing from the package suggest the mutilation occurred around the time of death/mummification.

Harnessing high-resolution non-destructive imaging for animal mummies provides new insights, exemplified by the visualization of features within the mummified snake package. Similar structures that appear to have been placed in the cobra’s mouth (Fig. 6e), have been seen in previous studies^63^, but their significance was unknown due to the low-resolution imaging. We have shown that in this case, these inclusions lie at the opening of the trachea, or the glottis. It has been suggested previously^8,16^ that the mouths of mummified snakes may have been filled with resin to render them harmless. For the first time, high-resolution imaging has enabled these structures to be visualized, located precisely, and identified (as probably natron). There are numerous possibilities for how these items are located at the glottis. The placement may have been an unintended consequence of the mummification process, which can include natron or similar materials. Alternatively, these items may have been placed in the mouth as part of an ‘opening of the mouth’ procedure. The latter is supported by the fact that the snake’s jaw is wide open, an unlikely final position without some intervention to prize open and maintain separation of upper and lower jaws. There is also clear trauma to the jaw bones and teeth, which has been observed in human mummies that have undergone the opening of the mouth procedure^66^; although this practice is previously undocumented in mummified snakes. If confirmed in other specimens, this could suggest that the mummification process for venomous snakes included complex ritualistic elements comparable to those described for the Apis Bull and human mummies. The papyrus Vienna 3,873^67^, includes a section detailing the preparation of the mouth, which includes the placement of myrrh and natron beneath the tongue of the bull as a desiccant, to retard decomposition. These structures are very small (3–4 mm), however, and the effect of desiccant would have been much more effective with large bags of natron or myrrh. It is perhaps possible that these items were precisely placed at the glottis by the people at that time, as described in the procedure for the Apis bull, on the throat openings.

The calcified kidneys identified by microCT provide insight into the life and death of this snake and the practices around animal mummification. Kidneys exhibiting calcification to this extent can be indicative of acute renal/kidney disease and gout, which has been seen in modern snakes and those kept as pets in poor conditions with little water^68,69^. This finding provides a glimpse into the past and the possible conditions in which this animal was kept, prior to its death, with the presence of crystals and tophi associated with gout eliciting a marked inflammatory response, which would have been painful to the animal.

There are several possible explanations for the absence of fangs in EC308. It is possible, given the potentially fatal potency of the venom, that they were removed to avoid post-mortem envenomation of the embalmers; venom can be potent long after death. The ancient Egyptians were aware of the effect of snake venom^70–72^, and working within the mouth around the sharp fangs may have been regarded as unsafe. It is also possible that these small structures became damaged and dislodged by the mummification process, particularly if the snake did undergo an opening of the mouth procedure.

Damage to the beak of the bird specimen (W531) and to the protruding foot mean that superficial visual identification of the bird is extremely difficult. However, microCT permits the measurement of bone elements in the correct plane, enabling us to identify the likely species of the mummy as a kestrel. Previous studies have shown that falcons has been found in mummified packages^5,13^, and birds of prey feature heavily in ancient Egyptian religion, although the exact species is not always identified by Egyptologists. Birds of prey are usually associated with solar gods, for example the gods Horus, Sokar, and Re. In a scene in the tomb Sennedjem, Deir-el-Medina, the sister goddesses Isis and Nephthys are depicted as divine mourners for the dead in the form of kestrels^73^. The ancient Egyptians would have been familiar with kestrels as they seem to have been the most frequently mummified raptor^57^. The large number of mummified wild birds suggests that many could have been collected from the wild rather than have been bred and nurtured in the temple precincts^8^. However, there is also clear evidence of breeding of certain animals^74^. Further interrogation of the imaging data enabled a thorough digital examination of the condition of the skeleton and revealed desiccated soft tissues and internal organs.

### Methodological reflections

We have presented a methodology and unique findings for high-resolution imaging of fragile and ancient specimens to reveal key morphometric structures and internal features. Traditional wisdom states that successful filtered back projection reconstruction of the 3D tomogram requires the entire sample width to be encompassed within each 2D projection or ‘field of view’ at all rotations^44^. This results in larger samples being imaged at lower resolutions. This is demonstrated in the initial scans of the whole snake specimen EC308, which captured the entire package while also providing sufficient contrast to reveal the internal snake skeleton when segmenting based on attenuation; yet the resolution was insufficient to resolve morphological features.

Using the ROI methodology, we were able to zoom into the skull and apply an offset rotation when acquiring the 2D projections. This method enabled a fivefold increase in resolution with no apparent degradation of the 3D data. The aim was to generate more detailed imaging of the skull morphology to aid species identification and investigate bone damage in that region. This was only made possible using ROI tomography. RoI imaging is not widely utilised with such specimens, with many users or researchers imaging samples based on traditional methods; maintaining the entire sample within the field of view. Using RoI could yield significant magnification and resolution improvements, ultimately enabling additional analyses and greater confidence in findings, improved measurement accuracy, and identification of smaller features.

Accurate measurements are also possible using 3D microCT data, because the measurement plane can be oriented and reoriented into a standard anatomical position. In contrast, measurements taken from 2D radiography, which will be inaccurate if the radiograph is not oriented parallel to the bone, particularly when measuring multiple bones of a skeleton from a small number of radiographs. 2D radiography also provides a projection through all of the contents, whereas digital slices taken from 3D tomograms show only the structures on the plane of the digital slice. The improved resolution of microCT compared to 2D radiography and medical CT improves the accuracy of both 2D and 3D measurements, and, depending on the resolution of the machine/scan, can be even more precise than methods using calipers on the actual bone.

## Methods

### Specific imaging protocols

3D geometric data for the range of specimens was collected on a Nikon XT H 225 microfocus X-ray tomography system (Nikon Metrology, Tring, UK) in the Advanced Imaging of Materials (AIM) Facility at the College of Engineering, Swansea University, UK. This is a laboratory-based X-ray system capable of imaging a broad range of materials, densities, and sizes. Images were captured with a Varian PaxScan 2,520 amorphous silicon flat panel digital X-ray imager, in reflection mode with either a molybdenum or tungsten target. The image acquisition parameters for the individual scans are presented in Supplementary Information Table S3.

The mummified cat body (AB77b) was too large to be imaged in one scan, therefore three scans were performed with a 20 mm vertical overlap, and the resulting 3D tomograms were stitched together in the visualisation software VGStudio Max 2.1.5 (Volume Graphics, Heidelberg, Germany). This produces a higher overall scan resolution/smaller voxel size by allowing the specimen to be imaged closer to the X-ray source. The resultant composite 3D tomogram is relatively large (22.5 GB), therefore requiring greater computational resource to both visualize and analyse than one smaller individual scan/tomogram, even on high-end visualization workstations. It is possible to sub-sample the data from 32/16 bit to 8 bit, although this compression can reduce density contrast^75^, meaning that the voxel intensities of different materials are more similar. This can cause problems when attempting to segment materials within the same tomogram that have similar densities/attenuation.

The bird (W531), was imaged in an upright position with the longest dimension oriented vertically in three separate overlapping scans with subsequent stitching of the resulting tomograms. The three separate scans were conducted using the same conditions, outlined in Supplementary Information Table S3.

**Figure 8:**
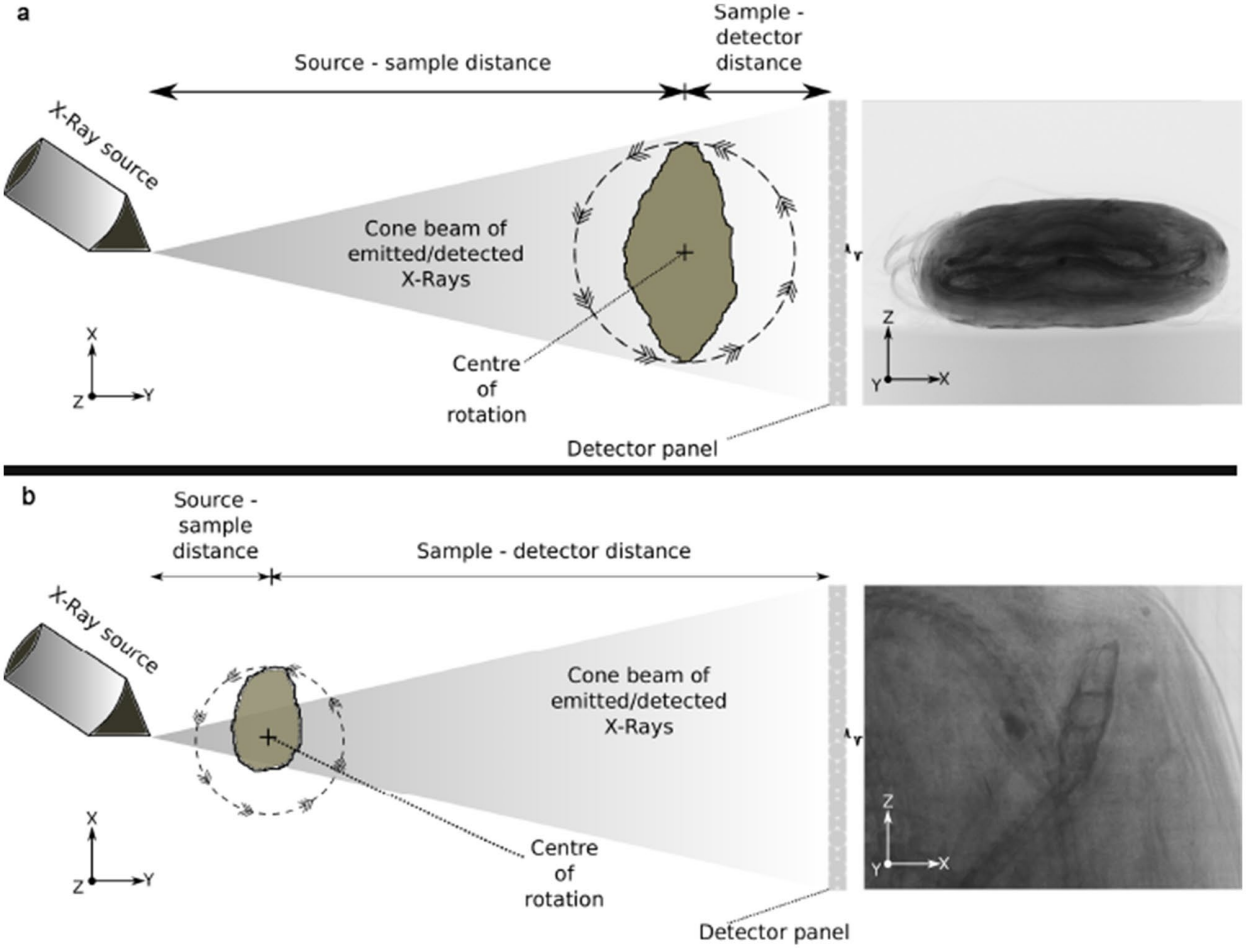
**a** Conventional setup for cone-beam X-ray microCT, with rotating sample completely within the field of view at all rotations and projections. The image on the right is an example of a 2D X-ray projection of the entire snake specimen, EC308. **b** Region of interest setup for cone beam X-ray microCT, with much smaller source-sample distance, resulting in much improved resolution of the final tomogram. The specimen is mounted vertically, and the field of view is focused on the snake skull in this example. Most of the specimen is rotated out of the field of view, but the skull is included in all projections.

The entire snake specimen (EC308) was imaged in one conventional scan, configuration (Fig. 8a), but the size of the mummified package compared to the skeletal remains resulted in small bones being imaged at a low-medium resolution (voxel size = 102 µm). From this low-resolution scan, the skull area was located and identified as a critical region for species identification. Therefore we employed a Region of Interest (RoI) tomography methodology^76^ to ‘zoom’ into the skull area at a higher resolution, ignoring the surrounding material; configuration shown in Fig. 8b. The initial whole specimen scan resulted in a voxel size of 102 µm, whereas the RoI scan resulted in a voxel size of 20.5 µm.

For each scan, a number of 2D projections, outlined in Supplementary Information Table S3, were gathered while the specimen was rotated 360°. The tomograms were reconstructed from the 2D projections using Nikon CTPro version 3.1.3 software (Nikon Metrology, Tring, UK). The commercial software VGStudio Max 2.1.5, the free software Drishti Version 2.4.4)^77^, and the Virtual Reality software Syglass Version 1.4.4 (IstoVisio, Inc. Morgantown, WV)^78^ were used to view the reconstructed data, 2D slices and rendered 3D volumes and produce Supplementary Information movie files.

By altering the voxel intensity threshold within the visualization software, it is possible to digitally dissect the remains, removing the wrappings based on their X-ray attenuation/greyscale value, which is distinct from the skeleton. With the external wrappings digitally removed, analysis of the bones and other internal anatomy is enabled.

## Conclusions

In this study we applied microtomography to the study of three Egyptian animal mummies. Application of this methodology provided insight into the life and death of these animals, mummification processes, and handling/storage in the following thousands of years. This can give valuable information on ancient Egyptian attitudes towards animals and the ancient Egyptian religion.

Microtomography produces much higher resolution than clinical CT scans and other non-destructive methods, enabling detailed features to be imaged in 3-dimensions. The use of ROI scanning also demonstrates the improvements in resolution possible on specimens such as mummified animals. This methodology has broad applicability to animal studies at high resolution, taking advantage of non-destructive, positional, ROI, morphometrics/measurement of bones at correct position and plane—X-ray imaging shedding a new light on the hidden and the mysterious. Occluding tissues and structures can be digitally removed to reveal previously obscured structures. The 3D data generated from microtomography can be exported for 3D printing, and structures can be scaled up, making the invisible visible, without damaging the delicate remains.

The application of these methods to an ancient mummified cat allowed identification of a juvenile cat (*F. catus*) that had been strangled. The mummified bird most closely resembles a Eurasian kestrel (*F. tinnunculus*). The oval specimen was identified as a mummified juvenile Egyptian Cobra (*Naja haje*), that had potentially been kept without sufficient fluids during its life, and ultimately killed by a whipping action, prior to undergoing an ‘opening of the mouth’ procedure during mummification.

## Supplementary information

Supplementary Movie 1.

Supplementary Tables.

Supplementary Movie 2.

Supplementary Movie 3.

Supplementary Movie 4.

Supplementary Movie 5.

Supplementary Movie 6.

Supplementary Movie 7.

Supplementary Movie 8.

Supplementary Movie 9.

## Data Availability

The bone measurement data is available in the Supplementary Material. X-ray microCT data that support the findings of this study are available from the following Zenodo DOIs: W531 Bird mummy—10.5281/zenodo.3856632, AB77a Cat mummy head—10.5281/zenodo.3856475, AB77b Cat mummy body—10.5281/zenodo.3857621, EC308 Snake mummy whole—10.5281/zenodo.3857257, EC308 Snake mummy head—10.5281/zenodo.3856408. Movie files for the specimens are available from: AB77a Cat mummy skull—10.6084/m9.figshare.12328367, AB77a Cat mummy skull2—10.6084/m9.figshare.12328301, AB77a Cat mummy mandible—10.6084/m9.figshare.12360674, AB77b Cat mummy body—10.6084/m9.figshare.12360677, AB77b Cat mummy body slices—10.6084/m9.figshare.12360680, W531 Bird mummy—10.6084/m9.figshare.12360683, W531 Bird mummy VR—10.6084/m9.figshare.12411023, EC308 Snake mummy whole—10.6084/m9.figshare.12360689, EC308 Snake mummy skull—10.6084/m9.figshare.12360695. STL files for 3D printing are available in figshare with the identifiers: Cat skull—10.6084/m9.figshare.9970301, Snake skull—10.6084/m9.figshare.9902372.
