# Supplementary material for: Evidence of diet, deification, and death within ancient Egyptian mummified animals
Source: Sci Rep. 2020 Aug 20;10:14113. doi: 10.1038/s41598-020-69726-0 (PMC7441183; doi:10.1038/s41598-020-69726-0)
Supplement: Supplementary file 2 — Supplementary Tables. [file 41598_2020_69726_MOESM2_ESM.pdf]

Supplementary Information for

Evidence of diet, deification, and death within ancient Egyptian mummified animals

Richard. Johnston*[1], Richard Thomas[2], Rhys Jones[3], Carolyn Graves-Brown[4], Wendy Goodridge[4], Laura North[1]

[1]Advanced Imaging of Materials (AIM) Facility, College of Engineering, Swansea University, Swansea, SA1 8EN, United Kingdom,

[2]School of Archaeology and Ancient History, University of Leicester, Leicester, LE1 7RH, United Kingdom

[3]School of Biosciences, Cardiff University, Cardiff, United Kingdom

[4]The Egypt Centre, Museum of Egyptian Antiquities, Swansea University, Swansea, SA2 8PP, United Kingdom

* Richard Johnston

Email: r.johnston@swansea.ac.uk

Other supplementary materials for this manuscript include the following:

Movies S1 to S9

Table S1. Means (and ranges) of measurements of *F. chaus*, *F. sylvestris* and *F. margarita* compared with AB77a. Comparative data from (42, 43).

	AB77a	*F. chaus nilotica*	*F. sylvestris libyca*	*F. margarita*	*F. catus*
Condyloincisive length (mm)	59.26	112.8 (98.2-123.9); n=18	83.1 (70.8-90.6); n=11	77.9 (n=1)	79.13 (69.0-91.2); n=60
Alveolar length (C-M_1) (mm)	24.46	39.6 (34.9-41.9); n=14	26.3 (26.1-31.7); n=12	-	23.66 (19.8-24.9); n=60

Table S2. Measured bone dimensions of W531 and reference species. Measurements are reproduced in millimeters. The humerus has been excluded because both humeri were fractured in W531. Where possible, measurements for males and females have been included because of the pronounced sexual dimorphism of raptors (females being larger than males). The specimen W531 was assessed against comparative specimens from the Solti (61) and the web resource by McNall (62).

Species	Sex	Tarsometatarsus (GL)		Tibiotarsus (GL)		Femur (GL)		Cranium (Greatest width)		Ulna (GL)		Radius (GL)		Carpometacarpus (GL)		Source
**W531**	Unknown	n=1	41.48	n=1	60.17	n=1	44.37	n=1	29.21	n=1	62.69	n=1	58.45	n=1	36.52	
Merlin	Male	n=10	34.9-38.4	n=10	52.6-58.9	n=10	**41.3-45.8**	n=10	26.4-27.1	n=9	49.8-52.9	n=10	45.2-51.3	n=11	**29.8-38.6**	Royal BC Museum
	Male	n=17	34.6-37.7	n=11	51.8-55.6	n=13	**41.9-45.1**	n=14	26.0-28.4	n=16	49.3-54.3	n=16	45.3-49.6	n=17	29.6-33.1	Solti (1996)
	Female	n=11	36.9-40.1	n=11	**57.3-61.8**	n=11	45.6-48.4	n=9	27.4-29.0	n=11	54.9-60.0	n=11	50.2-55.4	n=11	32.7-35.6	Royal BC Museum
	Female	n=20	36.6-40.7	n=6	**56.8–61.2**	n=11	**44.4-49.2**	n=19	26.8-28.8	n=21	53.8-60.4	n=21	49.3-55.6	n=21	**32.6-36.6**	Solti (1996)
Lanner Falcon	Male		42.5-55.0													Solti (1996)
Saker Falcon	Male		52.0-57.0													Solti (1996)
	Female		54.0-60.0													Solti (1996)
Eurasian kestrel	Male	n=13	**37.5-43.7**	n=13	**55.7-61.4**	n=21	**42.8-47.0**	n=14	**28.5-30.5**	n=17	**56.5-64.7**	n=17	**52.8-60.5**	n=14	**33.1-37.6**	Solti (1996)
	Female	n=23	**38.4-42.7**	n=25	**56.3-63.4**	n=26	**41.6-47.6**	n=22	**27.7-31.3**	n=25	**59.1-67.0**	n=25	**54.6-62.7**	n=25	**33.3-39.3**	Solti (1996)
Lesser Kestrel	Male	n=2	30.5-32.4	n=2	46.0-50.0	n=3	34.2-36.7	n=2	23.6-24.8	n=2	55.0-55.5	n=1	51.4	n=2	31.8-32.0	Solti (1996)
	Female	n=1	30.4	n=1	47.4	n=2	35.4	n=1	25	n=1	54.9	n=1	50.3	n=1	31.7	Solti (1996)
Hobby	Male	n=11	33.0-35.3	n=16	54.2-57.2	n=16	**40.8-44.9**	n=10	**27.5-29.7**	n=11	**57.4-63.5**	n=11	**53.6-58.6**	n=11	**34.9-38.5**	Solti (1996)
	Female	n=7	35.1-36.0	n=20	**57.9-60.6**	n=21	**43.5-46.8**	n=8	29.4-30.4	n=7	63.3-67.5	n=7	58.6-63.0	n=7	39.0-40.2	Solti (1996)
Eleanora's falcon	Unknown	n=0	no data	n=1	63.4	n=2	47.6-49.0	n=1	30.3	n=1	76.7	n=0	no data	n=0	no data	Solti (1996)
Red footed falcon	Male	n=10	27.4-30.7	n=9	46.4-49.7	n=11	35.0-38.4	n=9	24.8-26.9	n=10	54.6-60.2	n=10	50.6-55.8	n=10	31.5-34.2	Solti (1996)
	Female	n=13	27.2-30.9	n=14	45.6-52.0	n=14	35.1-38.9	n=12	24.2-27.0	n=14	54.5-60.6	n=13	50.5-54.9	n=14	31.4-34.6	Solti (1996)

Table S3. X-ray imaging acquisition parameters

Specimen	X-ray voltage (kV)	X-ray current (μA)	Filtration	Exposure time (ms)	Projections	Frames per projection	X-ray target material	Voxel size (μm)
**AB77a**	55	400	none	1415	3016	1	Tungsten	78.05
**AB77b**	55	400	none	1415	3016	1	Tungsten	65.22
**W531**	55	400	none	1415	1080	1	Molybdenum	59.04
**EC308 (low magnification)**	60	400	0.25mm Aluminium	1000	1080	4	Tungsten	102.09
**EC308 (ROI)**	85	274	none	1000	3016	1	Tungsten	20.5

Movie S1 (separate file). Digital dissection/unwrapping of the cat mummy AB77a head, revealing the skull. DOI: 10.6084/m9.figshare.12328367

Movie S2 (separate file). Digitally dissected skull of the mummified cat AB77a. DOI: 10.6084/m9.figshare.12328301

Movie S3 (separate file). Segmented mandible of the cat mummy, AB77a. Individual teeth segmented to reveal premolars P3 (green) and P4 (yellow), and the unerupted mandibular molars, M1 (red), within the alveolar crypt. DOI: 10.6084/m9.figshare.12360674

Movie S4 (separate file). Digital dissection/unwrapping of the cat mummy body, AB77b. DOI: 10.6084/m9.figshare.12360677

Movie S5 (separate file). X-ray microtomography slice video of the cat body, AB77b. DOI: 10.6084/m9.figshare.12360680

Movie S6 (separate file). Digital dissection/unwrapping of the bird mummy, W531. DOI: 10.6084/m9.figshare.12360683

Movie S7 (separate file). Digital dissection/unwrapping of the bird mummy W531 virtual reality view, W531. DOI: 10.6084/m9.figshare.12411023

Movie S8 (separate file). Digital dissection/unwrapping of the whole snake mummy EC308, revealing the coiled snake skeleton at lower resolution. DOI: 10.6084/m9.figshare.12360689

Movie S9 (separate file). Region-of-interest X-ray microCT scan, zoomed into the head of the snake, at higher resolution. Objects in the snake's mouth are visible, in addition to widespread skeletal damage, and the calcified kidneys. DOI: 10.6084/m9.figshare.12360695

References

1. S. Ikram, *The mummy in ancient Egypt: equipping the dead for eternity*, A. Dodson, Ed. (Thames & Hudson, 1998).
2. S. Pasquali, S. Ikram, S. Porcier, *Creatures of Earth, Water and Sky: essays on animals in ancient Egypt and Nubia* (Sidestone Press, 2019).
3. S. Ikram, *Death and burial in ancient Egypt* (Longman, 2003).
4. G. Gnudi, A. Volta, S. Manfredi, F. Ferri, R. Conversi, Radiological investigation of an over 2000-year-old Egyptian mummy of a cat. *J. Feline Med. Surg.* **14**, 292–294 (2012).
5. L. Morgan, S. McGovern-Huffman, P. French-Kreigh, Comparison of two falconid mummies from the Late Period of Ancient Egypt using noninvasive techniques. *J. Raptor Res.* **45**, 357–361 (2011).
6. E. Bleiberg, Y. Barbash, L. Bruno, *Soulful creatures: animal mummies in ancient Egypt* (Brooklyn Museum in association with D Giles Ltd, 2013).

7. S. Ikram, Speculations on the role of animal cults in the economy of Ancient Egypt. *Appriv. Sauvag. Wild CENiM 11* **3**, 211–228 (2015).

8. S. Ikram, Ed., *Divine creatures: animal mummies in ancient Egypt* (American University in Cairo Press, 2005).

9. J. H. Taylor, *Unwrapping a mummy: the life, death and embalming of Horemkenesi*, 1st University of Texas Press ed (University of Texas Press, 1996).

10. A. Lucas, *The Results of the Chemical Analysis of Materials from the Mummies Found in the Tomb of Amenhotep II* (A. Moures, 1908).

11. P. L. Armitage, J. Clutton-Brock, A radiological and histological investigation into the mummification of cats from Ancient Egypt. *J. Archaeol. Sci.* **8**, 185–196 (1981).

12. L. M. McKnight, *Imaging applied to animal mummification in ancient Egypt* (Archaeopress, 2010).

13. L. W. Morgan, S. McGovern-Hoffman, Noninvasive radiographic analysis of an Egyptian falcon mummy from the late period 664-332 BC. *J. Avian Biol.* **39**, 584–587 (2008).

14. W. M. Pahl, Radiography of an Egyptian "cat mummy", an example of the decadence of the animal worship in the late dynasties. *Ossa* **12**, 133–140 (1986).

15. , Egyptian Mummies: Radiological Atlas of the Collections in the National Museum of Antiquities in Leiden. *Radiology* **241**, 686–686 (2006).

16. Mathaf al-Misri., Salima. Ikram, Nasry. Iskander, Majlis al-A`l lil-Athar (Egypt), *Catalogue general of Egyptian antiquities in the Cairo Museum. Nos. 24048-24056, 29504-29903 (selected), 51084-51101, 61089. Nos. 24048-24056, 29504-29903 (selected), 51084-51101, 61089.* (Supreme Council of Antiquities Press, 2002).

17. A. H. Allam, Computed Tomographic assessment of atherosclerosis in Ancient Egyptian mummies. *JAMA* **302**, 2091 (2009).

18. T. Appelboom, J. Struyven, Medical imaging of the Peruvian mummy Rascar Capac. *Lancet* **354**, 2153–2155 (1999).

19. S. Atherton-Woolham, L. McKnight, C. Price, J. Adams, Imaging the gods: animal mummies from Tomb 3508, North Saqqara, Egypt. *Antiquity* **93**, 128–143 (2019).

20. C. Baldock, *et al.*, 3-D reconstruction of an ancient Egyptian mummy using X-ray computer tomography. *J. R. Soc. Med.* **87**, 806–808 (1994).

21. T. H. Falke, M. C. Zweypfenning-Snijders, R. C. Zweypfenning, A. E. James, Computed tomography of an ancient Egyptian cat. *J. Comput. Assist. Tomogr.* **11**, 745–747 (1987).

22. R. Gupta, Y. Markowitz, L. Berman, P. Chapman, High-resolution imaging of an ancient Egyptian mummified head: new insights into the mummification process. *AJNR Am. J. Neuroradiol.* **29**, 705–713 (2008).

23. H. Hoffman, P. A. Hudgins, Head and skull base features of nine Egyptian mummies: evaluation with high-resolution CT and reformation techniques. *AJR Am. J. Roentgenol.* **178**, 1367–1376 (2002).

24. H. Hoffman, W. E. Torres, R. D. Ernst, Paleoradiology: advanced CT in the evaluation of nine Egyptian mummies. *RadioGraphics* **22**, 377–385 (2002).

25. S. Hughes, R. Wright, M. Barry, Virtual reconstruction and morphological analysis of the cranium of an ancient Egyptian mummy. *Australas. Phys. Eng. Sci. Med.* **28**, 122–127 (2005).

26. C. Jackowski, S. Bolliger, M. J. Thali, Common and unexpected findings in mummies from Ancient Egypt and South America as revealed by CT. *RadioGraphics* **28**, 1477–1492 (2008).

27. J. Kieser, J. Dennison, D. Anson, T. Doyle, R. Laing, Spiral computed tomographic study of a pre-Ptolemaic Egyptian mummy. *Anthropol. Sci.* **112**, 91–96 (2004).

28. D. Magid, B. M. Bryan, R. A. Drebin, D. Ney, E. K. Fishman, Three-dimensional imaging of an Egyptian mummy. *Clin. Imaging* **13**, 239–240 (1989).

29. L. M. McKnight, J. E. Adams, A. Chamberlain, S. D. Atherton-Woolham, R. Bibb, Application of clinical imaging and 3D printing to the identification of anomalies in an ancient Egyptian animal mummy. *J. Archaeol. Sci. Rep.* **3**, 328–332 (2015).

30. L. M. McKnight, S. Atherton-Woolham, The evolution of imaging ancient Egyptian animal mummies at the University of Manchester, 1972–2014. *Mummies Magic Med. Anc. Egypt* (2016) (September 11, 2019).

31. A. H. Melcher, S. Holowka, M. Pharoah, P. K. Lewin, Non-invasive computed tomography and three-dimensional reconstruction of the dentition of a 2,800-year-old Egyptian mummy exhibiting extensive dental disease. *Am. J. Phys. Anthropol.* **103**, 329–340 (1997).

32. S. Panzer, *et al.*, Reconstructing the Life of an Unknown (ca. 500 Years-Old South American Inca) Mummy – Multidisciplinary Study of a Peruvian Inca Mummy Suggests Severe Chagas Disease and Ritual Homicide. *PLoS ONE* **9**, e89528 (2014).

33. K. Robson Brown, H. Wood, The utility of minimal CT scanning in the study of two Egyptian mummy heads. *Int. J. Osteoarchaeol.* **9**, 199–204 (1999).

34. F. J. Rühli, T. Böni, Radiological and physico- chemical analyses of an unusual post mortem arte- fact in an Egyptian mummy. *J. Paleopathol.* **12**, 63–71 (2000).

35. F. J. Rühli, T. Böni, Radiological aspects and interpretation of post-mortem artefacts in ancient Egyptian mummies from Swiss collections. *Int. J. Osteoarchaeol.* **10**, 153–157 (2000).

36. D. H. Shin, *et al.*, Radiological analysis on a mummy from a medieval tomb in Korea. *Ann. Anat. Anat. Anz. Off. Organ Anat. Ges.* **185**, 377–382 (2003).

37. A. D. Wade, *et al.*, Foodstuff placement in ibis mummies and the role of viscera in embalming. *J. Archaeol. Sci.* **39**, 1642–1647 (2012).

38. S. D. Atherton-Woolham, L. M. McKnight, Post-mortem restorations in ancient Egyptian animal mummies using imaging. *Pap. Anthropol.* **23**, 9–17 (2014).

39. S. Ikram, *et al.*, Fatal force-feeding or Gluttonous Gagging? The death of Kestrel SACHM 2575. *J. Archaeol. Sci.* **63**, 72–77 (2015).

40. A. D. Plessis, *et al.*, Three-dimensional model of an ancient Egyptian falcon mummy skeleton. *Rapid Prototyp. J.* (2015) https:/doi.org/10.1108/RPJ-09-2013-0089 (September 11, 2019).

41. J. Romell, *et al.*, Soft-Tissue Imaging in a Human Mummy: Propagation-based Phase-Contrast CT. *Radiology* **289**, 670–676 (2018).

42. E. Maire, P. J. Withers, Quantitative X-ray tomography. *Int. Mater. Rev.* **59**, 1–43 (2013).

43. R. A. Ketcham, W. D. Carlson, Acquisition, optimization and interpretation of X-ray computed tomographic imagery: applications to the geosciences. *Comput. Geosci.* **27**, 381–400 (2001).

44. A. C. Kak, M. Slaney, *Principles of computerized tomographic imaging* (IEEE Press, 1988).

45. J. Rueckel, M. Stockmar, F. Pfeiffer, J. Herzen, Spatial resolution characterization of a X-ray microCT system. *Appl. Radiat. Isot.* **94**, 230–234 (2014).

46. Ibrahim. Helmy, D. J. Osborn, *The contemporary land mammals of Egypt (including Sinai) / Dale J. Osborn, Ibrahim Helmy.* (Field Museum of Natural History, 1980) (April 16, 2015).

47. Z. Kratochvil, Das postkranialskelett der Wild- und Hauskatze (Felis silvestris und F. lybica f. catus). *Acta Sci. Nat. Acad. Sci. Bohemoslov. - Brno* **10**, 1–43 (1976).

48. D. R. Brothwell, E. S. Higgs, Silver, I. A., *Science in archaeology: a survey of progress and research*, Revised and enlarged ed (Thames & Hudson, 1969).

49. P. Orsini, P. Hennet, Anatomy of the Mouth and Teeth of the Cat. *Vet. Clin. North Am. Small Anim. Pract.* **22**, 1265–1277 (1992).

50. D. Fleming-Farrell, K. Michailidis, A. Karantanas, N. Roberts, E. F. Kranioti, Virtual assessment of perimortem and postmortem blunt force cranial trauma. *Forensic Sci. Int.* **229**, 162.e1-162.e6 (2013).

51. T. Crist, A. Washburn, H. Park, I. Hood, M. Hickey, "Cranial Bone Displacement as a Taphonomic Process in Potential Child Abuse Cases" in *Forensic Taphonomy*, W. Haglund, M. Sorg, Eds. (CRC Press, 1996) (April 16, 2015).

52. M. S. Ponce de León, C. P. Zollikofer, New evidence from Le Moustier 1: computer-assisted reconstruction and morphometry of the skull. *Anat. Rec.* **254**, 474–489 (1999).

53. C. P. E. Zollikofer, *et al.*, Virtual cranial reconstruction of Sahelanthropus tchadensis. *Nature* **434**, 755–759 (2005).

54. C. P. E. Zollikofer, M. S. Ponce De León, R. D. Martin, Computer-assisted paleoanthropology. *Evol. Anthropol. Issues News Rev.* **6**, 41–54 (1998).

55. R. N. Smith, Fusion of Ossification Centres in the Cat. *J. Small Anim. Pract.* **10**, 523–530 (1969).

56. A. von den Driesch, Munich, *A guide to the measurement of animal bones from archaeological sites* (Peabody Museum of Archaeology and Ethnology, Harvard University, 1976).

57. C. Gaillard, G. Daressy, *La faune momifiée de l'antique Égypte* (Impr. de l'Institut français d'archéologie orientale, 1905) (April 15, 2015).

58. J. Hanzak, Egyptian Mummies of animals in Czechoslovak collections. *Z. Für Ägyptische Sprache Altertumskunde* **104**, 86–88 (1977).

59. L. Lortet, C. Gaillard, *La faune momifiée de l'ancienne Egypte* (1908).

60. M. J. Nicholl, *Handlist of the birds of Egypt* (Government Press, 1919).

61. B. Solti, The Comparative Osteomorphological Study of the European Small-statured Falcons (Aves: Falconidae). *Folia Hist. Nat. Musei Matra.* **21**, 5–282 (1996).

62. M. McNall, Avian Osteology - Bird Bone Identification Guide. *R. Br. Columbia Mus. Can. - Avian Osteol. - Bird Bone Identif. Guide* (February 7, 2017).

63. T. Falke, "Radiology of ancient Egyptian mummified animals" in *Essays on Ancient Egypt in Honour of Herman Te Velde*, J. van Dijk, Ed. (Styx, 1997).

64. K. Szpakowska, Striking Cobra Spitting Fire. *Arch. Für Relig.* **14** (2013).

65. A. Piankoff, *The Tomb of Ramesses VI.: Plates, recorded by N. Rambova; photographed by L.F. Husson* (Pantheon Books, 1954).

66. R. Seiler, F. Rühli, "The Opening of the Mouth"—A New Perspective for an Ancient Egyptian Mummification Procedure. *Anat. Rec.* **298**, 1208–1216 (2015).

67. R. L. Vos, *The Apis embalming ritual: P. Vindob. 3873* (Uitgeverij Peeters en Departement Oriëntalistiek, 1993).

68. M. Belasco-Zeitz, G. W. Pye, R. E. Burns, A. P. Pessier, Clinical Challenge. *J. Zoo Wildl. Med.* **44**, 807–810 (2013).

69. H. A. Miller, Urinary diseases of reptiles: Pathophysiology and diagnosis. *Semin. Avian Exot. Pet Med.* **7**, 93–103 (1998).

70. J. F. Nunn, *Ancient Egyptian medicine* (University of Oklahoma Press, 1996).

71. G. Pinch, *A guide to the gods, goddesses and traditions of ancient Egypt.* (Oxford University Press, 2002).

72. R. H. Wilkinson, *The complete gods and goddesses of ancient Egypt* (Thames & Hudson, 2003).

73. P. F. Houlihan, *The birds of ancient Egypt* (Aris & Phillips, 1986).

74. S. Atherton, D. Brothwell, R. David, L. McKnight, A healed femoral fracture of Threskiornis aethiopicus (Sacred Ibis) from the Animal Cemetery at Abydos, Egypt. *Int. J. Paleopathol.* **2**, 45–47 (2012).

75. R. L. Abel, C. R. Laurini, M. Richter, A palaeobiologist's guide to 'virtual' micro-CT preparation. *Palaeontol. Electron.* **15** (2012).

76. A. Kyrieleis, V. Titarenko, M. Ibison, T. Connolley, P. J. Withers, Region-of-interest tomography using filtered backprojection: assessing the practical limits. *J. Microsc.* **241**, 69–82 (2011).

77. A. Limaye, Drishti: a volume exploration and presentation tool in (2012), pp. 85060X-85060X–9.

78. S. Pidhorskyi, M. Morehead, Q. Jones, G. Spirou, G. Doretto, syGlass: Interactive Exploration of Multidimensional Images Using Virtual Reality Head-mounted Displays. *ArXiv180408197 Cs* (2018) (June 2, 2020).
